# Reductive Stress of Sulfur-Containing Amino Acids within Proteins and Implication of Tandem Protein–Lipid Damage

**DOI:** 10.3390/ijms222312863

**Published:** 2021-11-28

**Authors:** Chryssostomos Chatgilialoglu, Carla Ferreri

**Affiliations:** 1 ISOF, Consiglio Nazionale delle Ricerche, 40129 Bologna, Italy; 2 Center of Advanced Technologies, Adam Mickiewicz University, 61-712 Poznań, Poland

**Keywords:** reductive stress, free radicals, gamma-radiolysis, hydrogen atom, hydrated electron, methionine, cystine, peptide/protein, biomimetic chemistry, tandem damage

## Abstract

Reductive radical stress represents the other side of the redox spectrum, less studied but equally important compared to oxidative stress. The reactivity of hydrogen atoms (H^•^) and hydrated electrons (e^–^_aq_) connected with peptides/proteins is summarized, focusing on the chemical transformations of methionine (Met) and cystine (CysS–SCys) residues into α-aminobutyric acid and alanine, respectively. Chemical and mechanistic aspects of desulfurization processes with formation of diffusible sulfur-centered radicals, such as methanethiyl (CH_3_S^•^) and sulfhydryl (HS^•^) radicals, are discussed. These findings are further applied to biomimetic radical chemistry, modeling the occurrence of tandem protein–lipid damages in proteo-liposomes and demonstrating that generation of sulfur-centered radicals from a variety of proteins is coupled with the cis–trans isomerization of unsaturated lipids in membranes. Recent applications to pharmaceutical and pharmacological contexts are described, evidencing novel perspectives in the stability of formulations and mode of action of drugs, respectively.

## 1. Reductive Stress

Redox homeostasis in biology represents the variety of endogenous adaptative mechanisms to preserve physiological processes in a cell/organism and balances two sides: oxidative stress and reductive stress [1]. Attention has been largely given to the former starting from the fact that most organisms live under aerobic conditions, and oxidative challenges can often occur and affect/modify biomolecules. The state-of-art in this field is regularly reviewed and, also recently, reviews have summarized the role of reactive oxygen species (ROS) to be themselves or to generate signaling molecules, involved in fundamental cellular functions [2,3].

On the other side of the redox spectrum, reductive stress has been less widely investigated [4,5], although this process is not less important than the oxidative one. Indeed, reductive stresses may affect cellular redox homeostasis, alter biomolecules and, eventually, determine specific pathological states [6]. In particular, under hypoxia conditions, cancer biology evidenced the key role of reductive stress [7]. Generally, reductive stress is associated with redox couples of NADH/NAD^+^, NADPH/NADP^+^ and GSH/GSSG, exploring the consequences due to exceeding reduced species, which are as harmful as oxidation products [8,9,10].

Nowadays research interest on various cellular and organ systems are directed toward the study of the delicate balance between reductive and oxidative signaling, and how it can become, for example, a vital factor in cancer cell homeostasis associated with cell proliferation, progression, and drug resistance [1]. Interests include the study of the balance created by enzymatic systems [5], those involved in the oxidative stress for generation of ROS or reactive nitrogen species (RNS), such as NAD (P)H oxidases (NOX) for the generation of superoxide radical anion (O_2_^•−^) and uncoupled nitric oxidases for the generation of nitric oxide (NO^•^), as well as those involved in the reductive environment created by electron exchange, as in the case of the mitochondrial electron transport chain.

In the frame of redox balance, the reactivity of free radicals, such as, for example, in the above-mentioned ROS/RNS production, is connected with the control given by antioxidant systems. Free radical species generated in the biological environment display a wide range of partition coefficients and rate constants, for example, going from the hydrophilic fast reactive hydroxyl radical (HO^•^) to the diffusible and slow reactive O_2_^•−^, and both factors have an influence on their effects [11]. In particular, electrons (e^−^) and hydrogen atoms (H^•^), which can derive from the interaction of water in the biological environment with ionizing radiations, have been the subject of research activity within various groups since the seventies [12,13,14]. In those times, the diffusible H^•^ was found to react selectively with thiols and sulfur-containing residues in peptides and proteins. The generation of solvated electrons without radiations was also connected with the reactivity of vitamins (E, C, carotenoids) [15]. Our group brought the reductive stress reactivity in models of biomimetic chemistry with generation of thiyl radical species [16,17], which are known to have a rich redox chemistry in nature [18].

In this review, we wish to give an overview of the redox balance from the reductive stress side, treating the chemistry associated with the reactions of H^•^ and e^−^_aq_ with sulfur-containing amino acids within peptides and proteins, and then delineating the information obtained from biomimetic models, including the interesting consequence of the tandem protein–lipid damage.

## 2. Transient Reductive Species Generated by γ-Radiolysis

Gamma-radiolysis of neutral water is the most direct method of generation of the primary reactive species e^−^_aq_, HO^•^, and H^•^, together with H^+^ and H_2_O_2_, as shown in Reaction 1, without using additives. The values in brackets represent the radiation chemical yields (G) in units of μmol J^−1^ [19]. The first three reactive species are expected to react very fast with peptides and proteins. The rate constants of the three reactive species with a few biomolecules of interest in this article are reported in Table 1.
H_2_O 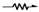 e^−^_aq_ (0.27), HO^•^ (0.28), H^•^ (0.06), H^+^ (0.27), H_2_O_2_ (0.07)(1)

To investigate the reactivity of reductive species (e^−^_aq_ and H^•^) and their consequences on protein structures, HO^•^ have to be scavenged. To achieve this, the most efficient procedure is based on the addition of *tert*-butanol (*t*-BuOH), which efficiently scavenges HO^•^ radicals (Reaction 2, *k*_2_ = 6.0 × 10^8^ M^−1^ s^−1^), whereas it reacts slowly with H^•^ atoms (Reaction 3, *k*_3_ = 1.7 × 10^5^ M^−1^ s^−1^). For example, by using an Ar-flushed aqueous solution containing 0.2 M *t*-BuOH and 1 × 10^−4^ M peptide/protein and assuming that HO^•^ reacts with peptide/protein with a rate constant of 2.4 × 10^10^ M^−1^ s^−1^, HO^•^ will be scavenged by *t*-BuOH 50 times faster than by peptide/protein.

HO^•^ + *t*-BuOH → (CH_3_)_2_C(OH)CH_2_^•^ + H_2_O (2)

H^•^ + *t*-BuOH → (CH_3_)_2_C(OH)CH_2_^•^ + H_2_ (3)

It is also possible to investigate the reactivity of H^•^ alone. In N_2_O-saturated solutions (~0.02 M of N_2_O), e^−^_aq_ are efficiently transformed into HO^•^ via Reaction 4 (*k*_4_ = 9.1 × 10^9^ M^−1^ s^−1^). Under these conditions, H^•^ and HO^•^ account for 10 and 90% of the reactive species, respectively. Therefore, by using N_2_O-saturated solutions in combination with *t*-BuOH as additive, the reactivity of H^•^ atoms can be investigated. Alternatively, electrons are known to be efficiently converted to H^•^ when they react with protons (H^+^) or dihydrogen phosphate anions (H_2_PO_4_^−^). In Ar-deoxygenated H_2_PO_4_^−^ (10 mM) aqueous solutions, e^−^_aq_ may be converted into H^•^ atoms (Reaction 5, *k*_5_ = 1.5 × 10^7^ M^−1^ s^−1^), depending on the pH and other additives.

e^−^_aq_ + N_2_O + H_2_O → HO^•^ + N_2_ + HO^−^ (4)

e^−^_aq_ + H_2_PO_4_^−^ → H^•^ + HPO_4_^2−^ (5)

## 3. The Reductive Desulfurization of Methionine (Met) and Cystine (CysS-SCys) Residues in Peptides/Proteins

**Scheme 1:**
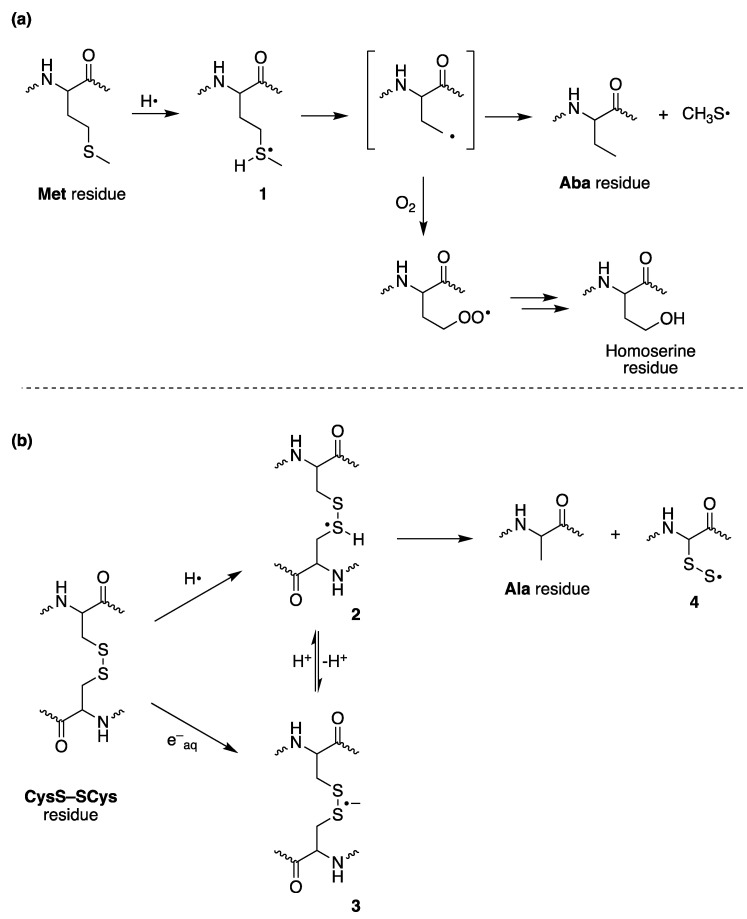
(**a**) Mechanism of reaction of H^•^ with Met residues in aqueous solution and the formation of α-aminobutyric acid (Aba) residue and diffusible CH_3_S^•^ radical. (**b**) Mechanism of reaction of H^•^ and e^−^_aq_ with CysS–SCys residues in aqueous solution and the formation of alanine (Ala) residue and perthiyl radical **4**.

A variety of sulfur-containing amino acids within peptides and proteins have been studied. The reactions of H^•^ with Met [21] and with various peptides containing a single Met residue, like Gly-Met-Gly [22], enkephalins [20] and amyloid-β [23] have been reported. The selective attack of H^•^ on sulfur atom of Met residues results in the formation of α-aminobutyric acid (Aba). Scheme 1a summarizes the proposed reaction mechanisms for these transformations. The desulfurization process of Met residue generates Aba and the diffusible methanethiyl radical (CH_3_S^•^) via the sulfuranyl adduct 1. The reaction **1** → Aba may be single or two-steps process with formation of CH_3_S^•^ and/or CH_3_SH. Indeed, in the γ-irradiation of the oxygenated aqueous solution of Met [21] or Gly-Met-Gly [22], Aba is replaced with homoserine which derives from the trapping of primary alkyl radical by oxygen, as shown in Scheme 1a. Pulse radiolysis studies on enkephalins indicated that Met residue is the main target of H-atoms [20]. In Met-enkephalin (Tyr-Gly-Gly-Phe-Met) the attack of H^•^ occurs about 50% on Met with formation of CH_3_S^•^ radical, and the remaining 50% is partitioned roughly evenly between Tyr and Phe. Experiments on amyloid-β peptide (Aβ) also showed the selective attack of H^•^ on the Met35 residue, with formation of a modified peptide containing an Aba residue [23].

Bovine pancreatic ribonuclease A (RNase A) and lysozyme (Lyso) are two medium-sized proteins that contain both Met residues and disulfide bridges with no free thiol group present. Early work on the inactivation of ribonuclease and ribonucleoside S-peptide by primary reactive species was reported [24,25,26]. In particular, the selective attack of H^•^ on CysS–SCys and Met residues resulted in the formation of alanine (Ala) and Aba (see Scheme 1). More recently, damage to bovine pancreatic RNase A, due to H^•^ and/or e^−^_aq_ attack at the protein sulfur-containing residues, was investigated by Raman spectroscopy and mass spectrometry techniques [27,28]. RNase A is a single polypeptide chain cross-linked by four disulfides bridges and contains also four Met. Indeed, reductive desulfurization process affects both Met and CysS–SCys residues. Mapping experiments demonstrated that desulfurization selectively affected Met79, Cys110, Cys58 and Cys72 during the first stages of reaction [28]. The concomitant occurrence of Met → Aba and CysS–SCys → Ala chemical mutations cannot be ascribed to modification processes in aqueous media other than radical-based desulfurization reactions, thus they are specifically attributed to reductive radical stress conditions. In the case of Lyso, a single polypeptide chain cross-linked by four disulfides bridges and contains two Met, the changes observed in the Raman spectra indicated that the degradation of sulfur moieties does not occur immediately, but as irradiation progresses [29]. Electron paramagnetic resonance (EPR) studies in the vacuum solid-state radiolysis of Lyso demonstrated that perthiyl radical (RSS^•^) residue is formed as the major product. In analogy with thiourea clathrate, where a head-to-head arrangement is imposed and there is no possibility of diffusible thiyl radicals, the equilibrium RS^•^ + RSH ⇆ RSS^•^ + (H)R can be followed by an irreversible conversion of sulfuranyl radical to perthiyl radical [30].

Scheme 1b summarizes the proposed reaction mechanisms for the reactions of H^•^ and e^−^_aq_ with CysS–SCys residues in proteins. The H^•^ attack gives rise to the sulfuranyl adduct **2**, which is in equilibrium with its deprotonated form, namely the disulfide radical anion **3**. The latter species may also be derived from the direct e^−^_aq_ attachment to the disulfide moiety. Both intermediates **2** and **3** dissociate reversibly into two entities, RS^•^/RSH and RS^−^/RSH respectively, but do not diffuse freely owing to the tertiary structure of the protein. Under these conditions, radical **2** undergoes irreversible decay with formation of Ala and perthiyl radical **4**. Radical anion **3** is also an excellent one-electron reducing agent [31].

**Figure 1:**
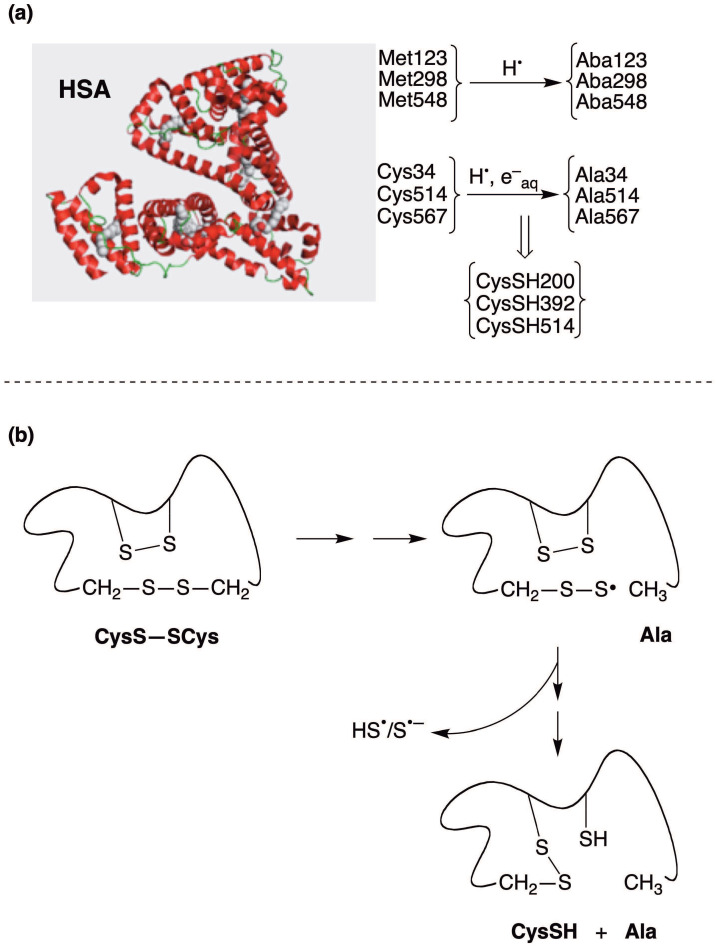
Human serum albumin (HSA): (**a**) Methionine (Met) and Cysteine (CysS–SCys) residues are modified by attack of reductive transient species (H-atoms or hydrated electrons), yielding α-aminobutyric acid (Aba) and alanine (Ala)/CysSH, respectively; (**b**) Proposed mechanism for the formation of alanine residues (cf. Scheme 1b) and CysSH derived from a scrambling event with other CysS–SCys.

Human serum albumin (HSA) is a large protein and accounts for over 50% of the total plasma protein content. HSA contains 6 Met and 35 Cys residues that form 17 CysS–SCys bridges and leave a unique thiol functionality that is involved in various in vivo HSA actions [32]. More than 70% of the total free radical-trapping activity of serum is due to HSA [33]. The reactivity of sulfur-containing amino acids in HSA under reductive radical stress conditions was also investigated by γ-radiolysis coupled with Raman spectroscopy and mass spectrometry techniques [34,35]. On the basis of the observed S–S stretching band changes, it was suggested that albumin disulfide bridges should eventually scramble upon reductive species attack, which can be correlated with secondary structure changes. MALDI-TOF and semiquantitative LC-ESI-IT-MS/MS peptide mapping experiments on different enzymatic digests demonstrated the following chemical mutations since the early stages of reaction: (i) Met123, Met298 and Met548 conversions into the corresponding Aba; (ii) the modification of Cys34, Cys514, and Cys567 in the cystine moieties into the corresponding Ala; and (iii) the formation of Cys200, Cys392 and Cys514 as free thiols of CysSH residues (Figure 1a). Based on the known cystine pairing in native albumin, no relationships were observed between desulfurization (formation of Ala residues) and the liberated cysteines (CysSH200, CysSH392, CysSH514). Figure 1b illustrates the suggested pathway where the CysS-S^•^ intermediate in the presence of another disulfide bridge of the molecule undergoes a quick disulfide scrambling event, generating the new free cysteine residues, the new disulfide bridge in a more stable secondary structure and the diffusible reactive sulfhydryl radical (HS^•^/S^•−^) responsible of the liposome interaction (see below) [34].

Metallothioneins (MTs) are sulfur-rich proteins capable of binding metal ions to give metal clusters. Metal–MT complexes are characterized by a high number of cysteine residues, the absence of disulfide bridges and the presence of labile sulfide anions (S^2−^) as non-protein (metal) ligands [36]. Reactions of reactive reductive species (H^•^ atoms and e^−^_aq_), produced by γ-irradiation of water, with Zn^II^–MT and Cd^II^–MT were carried out in aqueous solutions and monitored by various spectroscopic and spectrometric techniques (Raman, CD, and ESI-MS) [37,38,39]. The preferential attack site in both MTs is the Cys residues, as evidenced by measuring the intensity decrease in the Raman spectrum of some metal-S stretching bands. The modification of the metal–MT aggregates can induce significant structural changes, such as partial deconstruction and/or rearrangement of the metal clusters and breaking of the protein backbone. Substantial differences were observed in the behavior of the Zn^II^–MT and Cd^II^–MT aggregates towards the reactive species, depending on the different folding of the polypeptide in these two cases.

## 4. Biomimetic Radical Chemistry

### 4.1. Cis–Trans Isomerization of Fatty Acid Residues in Liposomes by Sulfur-Centered Radicals

Free-radical-catalyzed cis−trans isomerization of unsaturated lipids has been a subject of interest in our laboratory for the past two decades [40,41,42,43]. We demonstrated the reaction mechanism for this isomerization in various contexts, from simple reactions in various solvents to complex biomimetic models. We provided indications for an endogenous origin of trans unsaturated fatty acids in cells [44], animals [45], and humans [46,47] based on a mono-trans isomer of arachidonic acid as biomarkers [48,49].

**Figure 2:**
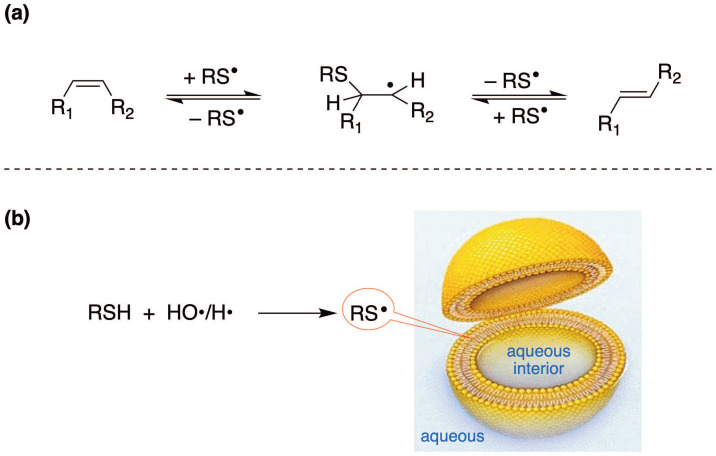
(**a**) Mechanism of thiyl radical catalyzed cis-trans double bond isomerization of unsaturated fatty acid moieties in phospholipids. (**b**) Thiyl radical generation in aqueous compartment and diffusion in the lipid compartment of large unilamellar vesicle (LUV) made of L-α-phosphatidylcholines (PC).

Τhe reaction mechanism of cis-trans isomerization is reported in Figure 2a and consists of a reversible addition of thiyl radical (RS^•^) to the double bond [43,50,51]. Indeed, the reconstitution of the double bond is obtained by β-elimination of RS^•^ and favors the trans geometry, which is the most thermodynamically favorable conformation. In this context, the radical RS^•^ acts as a catalyst for cis-trans isomerization, excluding the formation of positional isomers as reaction products because the mechanism does not allow a double-bond shift [43,51].

A simple biomimetic model of vesicle suspension demonstrated the potential efficiency of thiyl radicals (RS^•^) and their candidacy as culprits for the cis–trans isomerization in vivo. Liposomes can be represented as shown in Figure 2b, that is, a double layer formed by the spontaneous organization of phospholipid components in water, which delimits an aqueous cavity. Fatty acid tails can be saturated or unsaturated, and the disposition of the double bonds in the vesicle depends on the supramolecular arrangement of the bilayer. Unilamellar vesicles are the closest models of biological membranes and can be formed by different techniques, such as the extrusion and the injection methodologies. Studies of cis–trans isomerization of unsaturated moieties of DOPC (dioleoyl phosphatidyl choline) showed that the amphiphilic HOCH_2_CH_2_SH in LUVET (large unilamellar vesicle by extrusion technique) affords an efficient geometric isomerization, indicating that thiyl radicals generated in the aqueous compartment enter the hydrophobic region of the bilayer and isomerize the 9,10-double bond of oleic acid moieties.

The use of the biomimetic model of vesicle suspension demonstrated the potential of sulfhydryl radicals (HS^•^/S^•−^, p*K*_a_~4) derived from H_2_S to diffuse and reach the hydrophobic fatty acid chains, thus reacting with double bonds and producing trans-fatty acid containing phospholipids in high yield [52].

Experimental results supported the correlation of the origin of an endogenous formation of trans lipids with radical stress conditions that generate isomerizing reactive species [53,54,55,56,57]. However, the most feasible culprits for the lipid cis–trans isomerization in vivo are still a matter of discussion. Thiyl radicals RS^•^ are favored over other radicals [43,46], e.g., such as nitrogen centered radicals [53,54], on the basis of the efficiency of the thiyl radical-catalyzed cis−trans isomerization, demonstrated in vitro, and the presence of several sulfur-containing compounds in the cell, like peptides/proteins that contribute to the biological production of diffusible thiyl radicals.

### 4.2. Tandem Protein–Lipid Damage and Build-Up of Molecular Libraries

Tandem lesions are caused by a single radical event that leads to initial damage and, at the same time, produces reactive species able to damage another type of molecule. This tandem process involving lipids and proteins can involve two different cell compartments. Tandem damages are considered to be more harmful than single events since they can impair, or render more complicated, the cellular repair systems [58].

**Figure 3:**
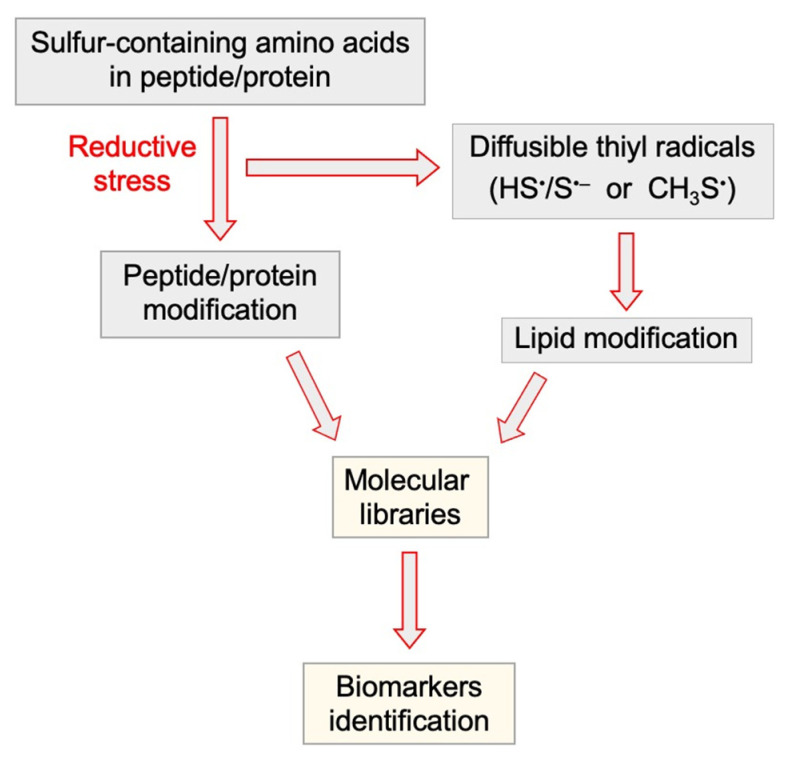
Concept of reductive stress and steps of the tandem protein–lipid damage leading to molecular libraries for biomarker identification.

Diffusible thiyl radicals generated by reductive stress of peptides or proteins are the best candidates to cause the isomerization of membrane unsaturated lipids. A radical reaction with the sulfur-containing amino acid as primary target will lead to modified proteins or peptides on one hand, and on the other hand to secondary diffusible radicals that can cause isomerization of membrane lipids. The modification of peptides/proteins and lipids will create molecular libraries to be implemented as potential biomarkers for reductive radical stress (Figure 3).

Biomimetic models were developed to study the process of lipid–protein damage. As described previously, ionizing radiation allows specific damage to be examined as a consequences of the reactivity of H-atoms and hydrated electrons, and this methodology was used to establish the first molecular basis of reductive radical stress. Based on the reductive desulfurization of Met and Cys residues described in Section 3, experiments carried out on proteo-liposome biomimetic models clearly showed that radical degradation of sulfur-containing residues produces sulfur-centered radical able to migrate into the hydrophobic part of the liposome and cause the radical-catalyzed isomerization of lipid double bonds.

**Figure 4:**
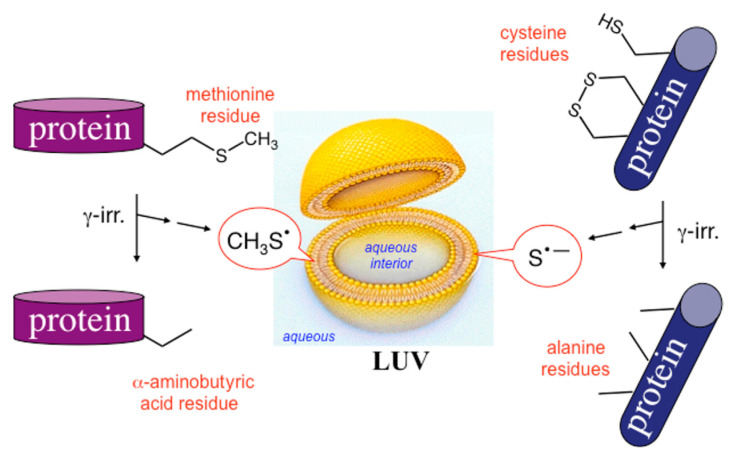
Tandem protein–lipid damage under reductive radical stress. Methionine and cystine residues are modified to α-aminobutyric acid and alanine/cysteine, respectively, by the attack of H^•^ and/or e^−^_aq_. Concomitant formation of diffusible sulfur-centered radicals (CH_3_S^•^ or S^•−^) able to migrate into the lipid bilayer induces cis−trans isomerization of unsaturated fatty acid residues. Taken from reference [43].

The model system of DOPC vesicles containing RNase A was the first example of tandem radical damage transferred from the protein to the lipid domain [17]. The proteo-liposome suspension (lipid:protein ratio ≥ 50:1) was exposed to the radical stress condition. To examine the fatty acid residues it is necessary to isolate phospholipids and perform the transesterification reaction in known conditions to obtain the corresponding fatty acid methyl esters (FAME). The FAME extract can be analyzed by gas chromatography (GC) which is the gold standard for the separation of geometrical and positional cis and trans isomers [43]. Figure 4 shows the various sulfur residues in proteins and the integrated vision of reductive radical stress which involves lipid modification and protein modification. The thiyl radical species formed from protein degradation can diffuse into the lipid bilayer and cause isomerization of the naturally occurring cis double bonds, using the mechanism shown in Figure 2a. The trans unsaturated fatty acids formed in vesicles result to be very sensitive markers of this protein damage, since the isomerization process is catalytic, therefore it can detect very low quantities of radicals generated in the environment. Protein damages were also determined by using Raman spectroscopy, to evidence changes in the secondary structures, such as, for example, in the case of RNase by detecting the 1230–1280 cm^−1^ region (amide III), which is conformationally susceptible, and indicated an increase in the percentage of β-sheet conformation of the protein [17]. Other analyses such as SDS-page demonstrated the formation of aggregates thus involving the tertiary structures of proteins, however, specific information on such changes were not achieved.

Based on the radical chemistry and modifications herein described, the development of trans fatty acid isomers as biomarkers of radical stress in vivo can be sustainable, with libraries developed for the recognition in biological samples. In particular, trans isomers not deriving from the diet, such as the trans isomer of sapienic acid (6trans-C16:1) and the 5trans and 8trans isomers of arachidonic acid (5cis,8cis,11cis,14cis-C20:4), are the best candidates to follow-up complex radical and redox balance in health and diseases [49,59,60].

As mentioned above and shown in Figure 1 and Figure 4, the model of palmitoyl oleoyl phosphatidyl choline (POPC) vesicles containing HSA showed the potential of proteins not containing free Cys moieties to produce diffusible sulfur radicals able to induce a cis–trans isomerization of lipids at the onset of irradiation [34]. This phenomenon led to focus our attention on the mechanism of the chemical modification of the sulfur-containing moieties (in particular, cystine) by reductive radical stress, as explained in Section 3. The modes of formation of S^•−^ species needs further comment, also in consideration that hydrogen sulfide (H_2_S) is a naturally occurring gas with roles in nervous and cardiovascular systems, and in pathological conditions, such as inflammation [61,62] and that perthiyl radicals are assuming more relevance in the frame of the thiyl radical reactivity and signaling events in biology [61].

From H_2_S the corresponding sulfhydryl radical (HS^•^) is generated, that has a p*K*_a_ value of ca. 4, therefore its deprotonated form S^•−^ dominates at neutral pH in an aqueous environment [52]. Indeed, using H_2_S generated in situ by its salt Na_2_S in the biomimetic model of vesicle suspension, we demonstrated the potential of sulfhydryl radicals (HS^•^/S^•−^) to induce efficiently the lipid cis–trans isomerization (cf. Figure 2b). When reductive radical stress conditions are used, the cystine residues can react, as summarized in Scheme 1b, to generate disulfide radical anions, derived from the direct electron attachment, that is generally in equilibrium with the sulfuranyl radical, also obtained by the direct H-atom attack on the sulfide moiety. The evolution of these intermediates is crucial since, as shown in Scheme 1b, the formation of alanine residue can be associated with the concomitant formation of perthiyl radical (RSS^•^). At this point, the question is: is the RSS^•^ radical an isomerizing species of unsaturated fatty acid residues? To our knowledge, no information exist on this reaction. The answer is probably negative based on the stability of this radical. Indeed, the bond dissociation energy of RSS–H is ca. 17 kcal/moL lower than the corresponding RS–H value [63]. There is evidence that *t*-BuSS^•^ adds reversibly to styrene where the benzyl-type adduct radical is stabilized by the phenyl ring [63].

Another question is: what is the fate of RSS^•^ radical? As shown in Figure 1a, the formation of Ala is associated with formation of CysSH. Based on the known cystine pairing in native albumin, no relationships were observed between desulfurization and formation of cystines, suggesting that the initially formed CysSS^•^ (see Figure 1b) undergoes a quick disulfide scrambling events toward more stable disulfide/thiol populations [34]. Based on the known data, we can propose that such scrambling events produce the observed CysSH (see Figure 1b) and HS^•^/S^•^^−^ species that catalyze the isomerization of double bonds.

It is worth also recalling the above reported proteo-liposome experiments on the reactivities of metallothioneins (MTs) under reductive stress. These proteins have different arrangements of metal–thiolate clusters formed by cysteine or histidine with endogenous sulfur content, and regulate the exogenous concentration of sulfide anions S^2−^ [36,37]. As mentioned above, substantial differences were observed in the behavior of the Zn^II^–MT and Cd^II^–MT aggregates towards the reductive reactive species (H^•^ and e^−^_aq_), due to different folding of the polypeptide in these two cases, using a biomimetic model based on unsaturated lipid vesicle suspensions [37,38,39,64]. Interestingly, Cd^II^-MT have a higher aptitude to induce cis–trans isomerization than Zn^II^–MT and this behavior is parallel with the number of acid-labile S^2−^ ligands, being higher in the former aggregate [37,43]. This suggests a major role of the S^2−^ present in the metal clusters as a precursor of diffusible isomerizing radicals like HS^•^/S^•−^ [52], although a different behavior due to the metal properties cannot be excluded.

To bring the tandem protein-lipid damage under radical stress conditions in an even larger context of molecular interactions, some interesting observations are reported for the reactivity of sulfur-containing drugs entrapped into liposome formulations. Besides being phospholipids the main components of cellular membranes, they have been developed as drug carriers. On the basis of the reactivity explained so far, and depending on the presence of the sulfur moieties on the molecular structures of the drug, it is interesting to evaluate the lipid cis-trans isomerization process and the outcome of radical stress conditions in two new perspectives, that are starting to be explored recently: (i) as a pharmaceutically relevant degradative process of the liposome formulation; (ii) as a pharmacologically relevant synergic process coupling the effect of the drug with the biological effect of the carrier containing trans lipids which are non-natural counterparts of the cis-lipids. It is worth mentioning that trans lipids have several adverse effects in biological health-related processes [65]. The entrapment of somatostatin, a natural peptide hormone that controls the cell growth, in a lecithin-based multilayer vesicle was obtained [66]. Somatostatin is a 14-amino acid containing molecule with two Cys residues linked by a disulfide bridge. The liposome formulation was seen to prolong its lifetime from 3 min to more than 24 h in human plasma. At the same time, when exposed to radical stress conditions, the formation of trans lipids in the liposome carrier was detected, clearly attributable to the formation of diffusible thiyl radicals from the reactivity of the disulfide bridge, as previously described [66]. In the frame of the drug mode of action, it is worth mentioning that, when drug activity involves complexes with metals and thiols in the biological environment reductive radical stress conditions can be created, as previously described for metallothioneins (see Section 3). This is the case of the anticancer drug bleomycin (BLM) and in vitro, it has been demonstrated that BLM-Fe^III^ complex with thiols is able to follow a reductive path of Fe^III^ to Fe^II^ with generation of RS^•^ and subsequent involvement of the membrane lipids in the cis-trans isomerization process [44]. A recent study of protein-lipid tandem damage showed that other amino acids of the molecular structure can trigger the formation of thiyl radicals by reductive stress and induce tandem damages. This is the case of a monoclonal antibody containing tryptophan moieties that, exposed to photoirradiation at 300 nm wavelength in the presence of Polysorbate-80, produces a tryptophan radical cation and a hydrated electron, which in its turn reduces a protein disulfide bond thus forming thiyl radicals and creating the lipid isomerization catalyst [67].

## 5. Conclusions

The evaluation of reductive radical stress in redox biology is still in its infancy compared with oxidative stress, despite the importance of the biomolecular transformations that such conditions can induce. The chemical basis of processes sustained by reductive species has been assessed, however, more investigation is needed especially concerning the monitoring of this reactivity under conditions of hypoxia, inflammation, or exposure to environmental, microbial and pharmacological agents, taking inspiration from the examples described in this review. Expanding such knowledge will also clarify aspects of the ongoing debate on the real advantages of antioxidant strategies, with the aim of an integrated vision of redox balance in various biological and pathological conditions.

## Figures and Tables

**Table 1:** Rate constant (M^−1^ s^−1^) of transient species derived from γ-radiolysis of water reacting with peptides/proteins [19].

Substrate	*k*(e^−^_aq_ )	*k*(H^•^)	*k*(HO^•^)
Methionine	4.0 × 10^7^	3.5 × 10^8^	8.3 × 10^9^
Glutathione disulfide	3.7 × 10^9^	1.0 × 10^10^	9.6 × 10^9^
Met-enkephalin ^1^	3.8 × 10^10^		
Ribonuclease	2.1 × 10^10^	1.5 × 10^10^	2.4 × 10^10^
Lysozyme	3.1 × 10^10^		4.9 × 10^10^
Serum albumin	8.2 × 10^9^		7.8 × 10^10^

^1^ From reference [20].
